# Mediation analysis of mental health characteristics linking social needs to life satisfaction among immigrants

**DOI:** 10.1016/j.ssmph.2023.101522

**Published:** 2023-10-04

**Authors:** David Adzrago, Faustine Williams

**Affiliations:** Division of Intramural Research, National Institute on Minority Health and Health Disparities, National Institutes of Health, Bethesda, MD, USA

**Keywords:** Quality of life, Well-being, Mental health, Immigration, Socioeconomics, Mediation analysis

## Abstract

**Background:**

Life satisfaction contributes to improved long and healthy lives, enhanced biological function, better mental health, and decreased mortality risks. Social needs (e.g., food security, employment, healthcare utilization) are important determinants of mental health and life satisfaction among immigrants. However, there is limited literature on how social needs influence mental health, which, in turn, affects life satisfaction among immigrants. We examined whether mental health influences the mechanisms of the relationship between social needs and life satisfaction among immigrants.

**Methods:**

We used the 2021 cross-sectional National Health Interview Survey data on U.S. immigrants (n = 4320) aged ≥18 years. We conducted weighted mediation analyses with multiple linear regression. Life satisfaction (scores 0–10; ≥1 as higher life satisfaction) was the dependent variable; independent variables were food security, employment, and healthcare utilization; and the mediator, serious psychological distress (SPD: scores 0–24; ≥1 as higher SPD).

**Results:**

The total effect (not accounting for SPD) of food insecurity (vs. secure) on life satisfaction was negative (β = −0.61, p < 0.001); the direct effect (after accounting for SPD) was not statistically significant (β = −0.21, p = 0.153), while the indirect effect (food insecurity's effect explained by SPD) was negative (β = −0.40, p < 0.001). The total (β = 0.32, p < 0.001), direct (β = 0.24, p = 0.004), and indirect (β = 0.09, p = 0.006) effects of being employed (vs. unemployed) on life satisfaction were positive. The total (β = −0.12, p = 0.116) and direct (β = −0.03, p = 0.683) effects of healthcare utilization within the past year (vs. more than a year) on life satisfaction were not statistically significant, whereas the indirect effect was negative (β = −0.09, p < 0.001).

**Conclusions:**

SPD mediates the effect of food security, healthcare utilization, and employment on life satisfaction, suggesting the need to improve social needs and mental health among immigrants.

## Highlights

• Mental health symptoms mediate the association between social needs and life satisfaction among immigrants in the US.
• Serious psychological distress (SPD) partially mediated the negative effect of food insecurity and past-year healthcare use on lower life satisfaction.
• The positive effect of employment on higher life satisfaction was reduced by about 26.60% after accounting for SPD.

## 1 Introduction

The world's largest immigrant populations (20%) live in the United States (U.S.) (Ward & Batalova, 2023). Immigrants represent about 13.6% (45.3 million) of the total U.S. population in 2021 (Ward & Batalova, 2023). They generally seek better socioeconomic (e.g., employment, income, food security) and safety (e.g., protection from forced displacement) opportunities in their host countries, including the U.S., that are often limited in their countries of origin (Berggren et al., 2020; Calvo et al., 2017). The satisfaction with their lives in the host country is a necessary determinant of their physical, emotional, social, and mental health (Berggren et al., 2020; Calvo et al., 2017). Life satisfaction, a key component of mental health (i.e., emotional, psychological, and social well-being), embodies individuals' evaluation of their perceived overall health and well-being (Ochiai et al., 2021), socioeconomic conditions (Organization for Economic Cooperation & Development [OECD], 2013), or richness, meaningfulness, and quality of their lives (American Psychological Association, 2023). Life satisfaction has been associated with health benefits such as longer and healthier lives and better mental health (Calvo et al., 2017, 2019; George, 2010; Kim et al., 2021). It also improves biological function by, for instance, enhancing inflammation levels and less hypertension (Blanchflower & Oswald, 2008; Hamer & Chida, 2011; Mojon-Azzi & Sousa-Poza, 2011; Uchino et al., 2018). Kim et al. (2021), for example, found in their nationally representative prospective study in the U.S. that life satisfaction improves physical, psychological, and behavioral health (Kim et al., 2021). Higher life satisfaction reduced 8%–46% risks of mortality, depression, physical functioning limitations, chronic pain, sleep problem onset, increased frequent physical activity, and decreased psychological well-being (e.g., negative affect, loneliness, hopelessness) (Kim et al., 2021). Despite the health benefits of life satisfaction, research on life satisfaction among immigrant populations in the U.S. is limited. Evaluating life satisfaction among immigrants will help determine their life satisfaction or its associated factors to provide tailored interventions to boost these populations' life satisfaction.

Previous studies have attributed socioeconomic and/or social needs and mental health factors to life satisfaction among immigrants. Social needs in the context of immigrants refer to complex, multifaceted necessary needs that an immigrant requires to survive or have healthy and productive lives in their host country (Artiga & Pham, 2019; El Arab et al., 2023; Hacker & Houry, 2022). These needs, including food security, employment, and healthcare utilization, significantly impact the life satisfaction of individuals, including immigrants (Chen & Hou, 2019; Chilton & Booth, 2007; Hacker & Houry, 2022; Kaiser et al., 2007; Pinard et al., 2015; Pourmotabbed et al., 2020; Salahodjaev & Mirziyoyeva, 2021). These social needs are important particularly for immigrants to survive or maintain their health and well-being in their host country because immigrants need food to survive, jobs to afford services, or medical care to improve their health (Artiga & Pham, 2019; El Arab et al., 2023; Hacker & Houry, 2022). Populations with higher food security or employment have higher life satisfaction (Chen & Hou, 2019; Salahodjaev & Mirziyoyeva, 2021). Food insecurity increases the risks of health outcomes and poor mental health (e.g., anxiety and depression) (Pourmotabbed et al., 2020). However, access to healthcare utilization decreases life satisfaction (Habibov & Afandi, 2016; Harrelson, 2019; Kim et al., 2014). Mental health has been associated with food security, healthcare utilization, and employment such that access to food, healthcare, or employment improves individuals’ mental health (Chen & Hou, 2019; Guo & Cheng, 2017; Pourmotabbed et al., 2020). Mental health is also a critical determinant of life satisfaction; people with poor mental health have low life satisfaction (Lombardo et al., 2018). The postmigration circumstances (e.g., acculturative stress, employment, cultural and linguistic barriers) experienced by immigrants often affect their mental health and quality of life (Derr, 2016; Noh & Kaspar, 2003). For instance, acculturative stress, a term used to describe postmigration challenges such as limited access to healthcare, jobs, and housing, has been linked to higher mental health problems, including psychological distress (i.e., anxiety and depression) (Baeza-Rivera et al., 2022; Derr, 2016). Immigrants who are unemployed or lack food tend to have high burdens of psychological distress such as anxiety and depression (Dou et al., 2022; Kirmayer et al., 2011; Shekunov, 2017; Walther et al., 2020). Higher levels of psychological distress increase the risks of lower life satisfaction among immigrants (Walther et al., 2020). Nonetheless, there is limited literature on how social needs impact mental health (including psychological distress) and life satisfaction, and the subsequent influence of mental health on life satisfaction among immigrants in the U.S. No study to date examined whether mental health influences the mechanisms of the relationship between social needs and life satisfaction among immigrants in the U.S. Most of the existing studies on life satisfaction among immigrants in the U.S. were conducted among older adults (Calvo et al., 2017, 2019; Harrelson, 2019; Kim et al., 2021; Park et al., 2012), yet about 95% of the immigrant population comprises those aged 18 years or more, and 77% working age group (18–64 years) (Ward & Batalova, 2023).

Due to better socioeconomic expectations of immigrants in their host country, evaluating their life satisfaction in the host country is necessary. Their socioeconomic conditions or social needs may impact their mental health, which may also impact their life satisfaction. However, the pathways linking social needs to life satisfaction among immigrants are less examined. While previous studies indicated that social needs are associated with mental health (Chen & Hou, 2019; Guo & Cheng, 2017; Pourmotabbed et al., 2020) and life satisfaction (Chen & Hou, 2019; Salahodjaev & Mirziyoyeva, 2021) and mental health with life satisfaction (Lombardo et al., 2018), it is unclear whether social needs directly or indirectly influence life satisfaction among immigrants. Thus, there is sparse information on whether social needs affect mental health, which in turn, influences life satisfaction. Understanding these pathways will help effectively improve immigrants' life satisfaction by influencing the pathways. Mediation analysis is an effective statistical method widely used to evaluate the pathways linking public health factors (Jung, 2021; Rijnhart et al., 2021). The mediation analysis estimates the direct and indirect pathways linking the factors. Hence, we conduct a mediation analysis to determine the mediation effects of mental health on the relationship between social needs and life satisfaction among a nationally representative sample of immigrant adults (18+ years) in the U.S.

## 2 Methods

### 2.1 Study design and population

We analyzed data from the 2021 National Health Interview Survey (2021 NHIS) deidentified public-use dataset. The National Center for Health Statistics (NCHS) contracted and sponsored the U.S. Census Bureau to conduct this nationally representative annual household cross-sectional survey between January and December 2021. NHIS measures health behaviors, healthcare service utilization, mental health, and socioeconomic characteristics of the civilian noninstitutionalized U.S. population. The sample includes individuals who reside in the 50 states and the District of Columbia at the time of the interview. The U.S. Census Bureau collects the NHIS data among the individuals living in the U.S. NHIS is a complex survey design where houses are systematically selected from counties within state boundaries, and an individual is randomly chosen from each household. The 2021 NHIS public-use data consist of 29,482 adults with a response rate of 50.9%. We focused our analysis on foreign-born adults (i.e., persons not born in U.S. or U.S. territory [n = 4709]) with complete data (n = 4320). Thus, foreign-born status was determined in NHIS by asking the participants whether (Yes/No) they were born in the U.S. or a U.S. territory: those who were not born in the U.S., or a U.S. territory were considered foreign-born/immigrant; otherwise, they were considered U.S.-born/non-immigrant.

### 2.2 Measures

#### 2.2.1 Primary dependent variable

Life satisfaction was examined as the dependent variable. This variable was measured with a single item, “Using a scale of 0–10, where 0 means ‘very dissatisfied’ and 10 means ‘very satisfied,’ how do you feel about your life as a whole these days?” The total scores range from 0 to 10, with higher values indicating higher life satisfaction with socioeconomic conditions (OECD, 2013), richness, meaningfulness, quality of life (American Psychological Association, 2023), or overall health and well-being (Ochiai et al., 2021).

#### 2.2.2 Mediator/secondary dependent variable

Serious psychological distress (SPD), a mental health or illness measure, was measured with a self-reported six-item Kessler scale (Kessler 6 scale). The participants were asked how often they feel sad, nervous, restless, hopeless, difficult effort, and worthless during the past 30 days. The response options include 1 = all the time, 2 = most of the time, 3 = some of the time, 4 = a little of the time, and 5 = none of the time. The response levels were reversed to obtain continuous total scores ranging from 0 to 24 for the Kessler 6 scale. Following Jann's STATA approach to estimate Cronbach's alpha (i.e., α) for weighted data (Jann, 2004), we computed the weighted test of reliability or internal consistency for the SPD based on the Kessler-6 scale and found high internal consistency for the scale (α = 0.870) with an average inter-item correlation of 0.5280.

#### 2.2.3 Independent variable

We examined food security, employment (employed or unemployed), and healthcare service utilization as the main independent variables. We used the food security status in the past 30 days described in NHIS as 1 = food secure, 2 = low food security, and 3 = very low food security. We recategorized the responses as food secure (1 = food secure) and insecure (2 = low food security, 3 = very low food security). The food security status measures the families' or households’ food situation. Healthcare utilization status was determined by asking the participants about the last time they saw a doctor or other health professional about their health. The participants were asked to select their options: 0 = never, 1 = within the past year (anytime less than 12 months ago), 2 = within the last 2 years, 3 = within the last 3 years, 4 = within the last 5 years, 5 = within the last 10 years, 6 = 10 years ago or more. We dichotomized this variable as utilized healthcare within the past year (1 = within the past year) and within the last 3 years or more/never used (options 0, 2–6).

#### 2.2.4 Confounders/covariates

Based on previous studies (Berggren et al., 2020; Calvo et al., 2017, 2019; Chen & Hou, 2019; Derr, 2016; Harrelson, 2019; Kim et al., 2021; Park et al., 2012; Ward & Batalova, 2023), we controlled for potential confounders such as sociodemographic characteristics: age (18–25, 26–34, 35–49, 50–64, or 65+), sex (male or female), sexual orientation (heterosexual, lesbian/gay, bisexual, or other [something else, or uncertain]), citizenship status (citizen or non-citizen), race/ethnicity (Hispanic, other race/ethnic group [non-Hispanic Asian, American Indian or Alaska Native, Native Hawaiian or other Pacific Islander, or other single and multiple races], non-Hispanic White only, non-Hispanic Black/African American only), and family income to poverty ratio (0.00–11+). We also adjusted for social/emotional support frequency, which was based on how often the participants receive the social and emotional support they need (response option: always, usually, sometimes, rarely, or never). We recoded the social/emotional support frequency into two groups: (1) always/usually/sometimes and (2) rarely/never.

### 2.3 Statistical analyses

All statistical analyses were performed using the NHIS 2021 sampling weights to offset nonresponse and compute nationally representative estimates. We estimated descriptive and bivariate statistics of the mean life satisfaction scores across the independent variables (food security, employment status, and healthcare utilization), mediator (SPD), and confounding variables (age, biological sex, sexual orientation, citizenship status, race/ethnicity, family income to poverty ratio, and social/emotional support frequency) (Table 1). We conducted the bivariate statistics with a *t*-test or ANOVA to determine differences in the mean life satisfaction scores based on the independent variables, mediator, and confounding variables. The statistically significant group differences were determined at an alpha level of ≤0.005 to minimize uncertainty in the significance of the group differences when weighted analysis or large data analysis is performed. We used STATA 17.0 to conduct our analyses.

**Table 1:** Differences in life satisfaction mean scores by sociodemographic characteristics, mental health, and social needs among immigrant adults (Unweighted n = 4320 and weighted N = 41,126,775).

	Overall sample	Life satisfaction	
	n%	M (SD)	P-value
**Overall**		8.424 (1.724)	
**Age groups**			<0.005
18–25	228 (8.10)	8.372 (1.525)	
26–34	619 (15.20)	8.563 (1.523)	
35–49	1414 (32.70)	8.571 (1.570)	
50–64	1168 (26.80)	8.312 (1.762)	
65 and older	891 (17.20)	8.218 (2.146)	
**Sex**			0.867
Male	1922 (47.83)	8.430 (1.655)	
Female	2398 (52.17)	8.418 (1.787)	
**Sexual orientation**			0.034
Heterosexual	4153 (96.09)	8.442 (1.714)	
Gay/Lesbian	52 (1.05)	8.144 (1.658)	
Bisexual	29 (0.63)	7.426 (2.218)	
Other/uncertain	86 (2.23)	8.053 (1.873)	
**Citizenship status**			0.303
Citizen	2658 (57.99)	8.393 (1.752)	
Non-citizen	1662 (42.01)	8.466 (1.679)	
**Race/ethnicity**			<0.005
Non-H White only	913 (17.90)	8.227 (1.813)	
Non-H Black/African American only	363 (9.82)	8.413 (1.579)	
Hispanic	1702 (45.34)	8.591 (1.653)	
Other/Multi-racial group	1342 (26.95)	8.278 (1.777)	
**Family income to poverty ratio (M [SD])**	3.638 (2.929)	0.023*	0.183
**Social/emotional support frequency**			<0.005
Always/usually/sometimes	3802 (88.53)	8.464 (1.652)	
Rarely/never	518 (11.47)	8.115 (2.202)	
**Employment status**			<0.005
Unemployed	1598 (35.53)	8.180 (2.023)	
Employed	2722 (64.47)	8.558 (1.528)	
**Healthcare utilization**			<0.005
Within the last two years or more/never used	940 (22.81)	8.558 (1.556)	
Within the past year	3380 (77.19)	8.384 (1.771)	
**Food security status**			<0.005
Secure	4001 (92.27)	8.473 (1.660)	
Insecure	319 (7.73)	7.836 (2.258)	
**SPD (M [SD])**	1.998 (3.456)	-0.391**	<0.005

*Standardized correlation coefficient (unstandardized correlation coefficient = 0.115, p = 0.189).

**Standardized correlation coefficient (unstandardized correlation coefficient = −2.331, p < 0.005).

Non-H = non-Hispanic. SD = standard deviation. M = mean. Weighted N = 41,126,775 and unweighted n = 4320.

**Fig. 1:**
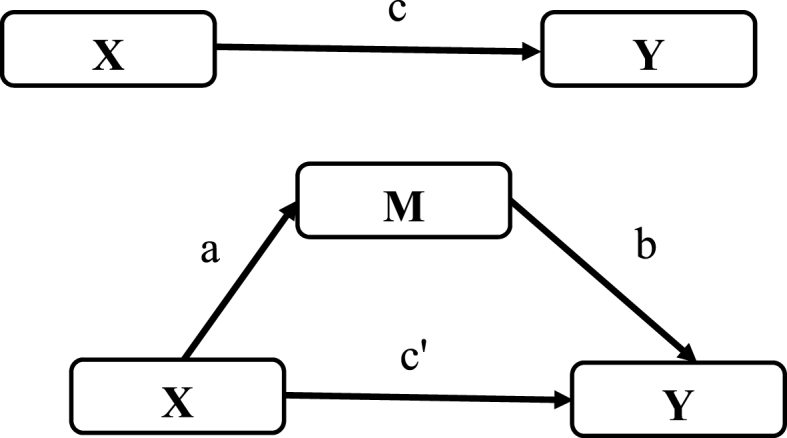
Statistical diagram of mediation. The top diagram represents the total effect model, and the bottom diagram illustrates the mediation model. Y = dependent variable, X = independent variable, M = mediator, a = effect of X on M, b = effect of M on X, c = total effect of X on Y, and c' = Direct effect of X on Y.

Mediation models were used to evaluate whether SPD mediates the relationship between food security, employment status, or healthcare utilization and life satisfaction, adjusting for the confounders or covariates. We evaluated the degree of multicollinearity of the independent variables and found a mean variance inflation factor (VIF) of 1.69, which is lower than the threshold (VIF = ≥ 10) to suggest significant multicollinearity (O'brien, 2007; Vittinghoff et al., 2006). Fig. 1 illustrates the statistical diagram of the mediation or path. We followed the approaches of Baron and Kenny (1986), Preacher and Hayes (2004), and Zhao et al. (2010) to perform the mediation analysis. The approaches consist of three regression models (X → Y, X → M, and X + M → Y) suggesting that (1) the independent variable significantly influences the mediator, (2) the independent variable significantly influences the dependent variable in the absence of the mediator, (3) the mediator has a significant effect on the dependent variable, and (4) the effect of the independent variable on the dependent variable shrinks upon adjusting for the mediator in the model. The approach requires a significant effect of the independent variable on the mediator to proceed with the mediation analysis. Three main mediation models were evaluated: Models A, B, and C. Model A examined whether SPD mediates the relationship between food security and life satisfaction (Table 2); Model B assessed whether SPD mediates the relationship between employment status and life satisfaction (Table 3); and Model C examined whether SPD mediates the relationship between healthcare utilization and life satisfaction (Table 4). We adjusted for the confounders (see the supplemental files for their results) in all the models. In each of the three main models, three sub-models (see the below equations) were evaluated to determine the independent variables' indirect, direct, and total effects on the dependent variable.

Model 1: Y_1_ = β_0_ + β_1_X + Z_i_ + ε

Model 2: M = β_0_ + β_1_X + Z_i_ + ε

Model 3: Y_1_ = β_0_ + β_1_X + β_2_M + Z_i_ + ε

Y is the dependent variable, X denotes the independent variable, M represents the mediator, Z represents the covariates/confounders, ε indicates the error term, β_0_ is the intercept, and β_1_ is the slope of the independent variable.

**Table 2:** Mediation effects of SPD on the relationship between food security and life satisfaction, adjusting for employment, healthcare utilization, and sociodemographic characteristics among immigrant adults (N = 4320).

	Effect	SPD (M)		Life satisfaction (Y)	
		Coeff. (SE)	95% CI	Coeff. (SE)	95% CI
**Food security status (Insecure vs. Secure)**	X				
R^2^		0.063		0.176	
Effect of X on M	Path a (X → M)	2.106*** (0.365)	(1.389, 2.823)		
Effect of M on Y	Path b (M → Y)			-0.190*** (0.014)	(-0.218, −0.162)
Indirect effect of X on Y	a*b (c - c')			-0.400*** (0.067)	(-0.528, −0.278)
Direct effect of X on Y	c'			-0.212 (0.148)	(-0.506, 0.082)
Total effect of X on Y	c (a*b + c')			-0.612*** (0.162)	(-0.939, −0.298)

Regression coefficients are unstandardized. Standard errors (SEs) are in parentheses. Bootstrap sample size = 1000. 95% CI = confidence interval. Coeff. = regression coefficient. Ref = reference group. *p < 0.05. **p < 0.01. ***p < 0.001. SPD = serious psychological distress. R^2^=R-squared.

**Note**: Proportion of total effect that is mediated: **0.654** (i.e., −0.400/−0.612 = 0.6536). The effect of food security is reduced by about 65.40% after accounting for SPD. Ratio of indirect to direct effect: **1.890.** Ratio of total to direct effect: **2.890.**

**Table 3:** Mediation effects of SPD on the relationship between employment and life satisfaction, adjusting for food security, healthcare utilization, and sociodemographic characteristics among immigrant adults (N = 4320).

	Effect	SPD (M)		Life satisfaction (Y)	
		Coeff. (SE)	95% CI	Coeff. (SE)	95% CI
**Employment status (Employed vs. unemployed)**	X				
R^2^		0.063		0.176	
Effect of X on M	Path a (X → M)	-0.453** (0.167)	(-0.781, −0.124)		
Effect of M on Y	Path b (M → Y)			-0.190*** (0.014)	(-0.218, −0.162)
Indirect effect of X on Y	a*b (c - c')			0.086** (0.032)	(0.021, 0.150)
Direct effect of X on Y	c'			0.237** (0.084)	(0.069, 0.396)
Total effect of X on Y	c (a*b + c')			0.323*** (0.089)	(0.150, 0.496)

Regression coefficients are unstandardized. Standard errors (SEs) are in parentheses. Bootstrap sample size = 1000. 95% CI = confidence interval. Coeff. = regression coefficient. Ref = reference group. *p < 0.05. **p < 0.01. ***p < 0.001. SPD = serious psychological distress. R^2^=R-squared.

**Note**: Proportion of total effect that is mediated: **0.266.** Ratio of indirect to direct effect: **0.363.** Ratio of total to direct effect: **1.363.**

**Table 4:** Mediation effects of SPD on the relationship between healthcare utilization and life satisfaction, adjusting for food security, employment, and sociodemographic characteristics among immigrant adults (N = 4320).

	Effect	SPD (M)		Life satisfaction (Y)	
		Coeff. (SE)	95% CI	Coeff. (SE)	95% CI
**Healthcare utilization (Used within the past year vs. within the last two years or more/never used)**	X				
R^2^		0.063		0.176	
Effect of X on M	Path a (X → M)	0.500*** (0.138)	(0.229, 0.770)		
Effect of M on Y	Path b (M → Y)			-0.190*** (0.014)	(-0.218, −0.162)
Indirect effect of X on Y	a*b (c - c')			-0.095** (0.028)	(-0.148, −0.037)
Direct effect of X on Y	c'			-0.029 (0.073)	(-0.171, 0.111)
Total effect of X on Y	c (a*b + c')			-0.124 (0.076)	(-0.277, 0.022)

Regression coefficients are unstandardized. Standard errors (SEs) are in parentheses. Bootstrap sample size = 1000. 95% CI = confidence interval. Coeff. = regression coefficient. Ref = reference group. *p < 0.05. **p < 0.01. ***p < 0.001. SPD = serious psychological distress. R^2^=R-squared.

**Note:** Proportion of total effect that is mediated: **0.765.** Ratio of indirect to direct effect: **3.263.** Ratio of total to direct effect: **4.263.**

Based on recommendations by Preacher and Hayes (2008) and Zhao et al. (2010) in obtaining mediation estimates with high statistical power, we used bootstrapping with 1000 replications to obtain the indices of the mediation with their 95% confidence intervals (CIs), standard errors (SEs), and p-values.

## 3 Results

In Table 1, the highest proportion of the immigrant population aged 35-49 (32.70%), were females (52.17%), identified as heterosexual (96.09%), citizens (57.99%), or Hispanics (45.34%). Most of them had a mean family income to poverty ratio of 3.638 (SD = 2.929), had social/emotional support always/usually/sometimes (88.53%), employed (64.47%), used healthcare within the past year (77.19%), and had food security (92.27%). The average SPD score was 1.998 (SD = 3.456), and the average life satisfaction score was 8.424 (SD = 1.724). The bivariate statistics showed that the lowest average life satisfaction scores were among those aged 65 years or more, identified as bisexual, non-Hispanic White only persons, rarely or never received social/emotional support, employed, used healthcare within the past year, and experienced food insecurity. There was a negative correlation between SPD and life satisfaction (r = −0.391).

**Fig. 2:**
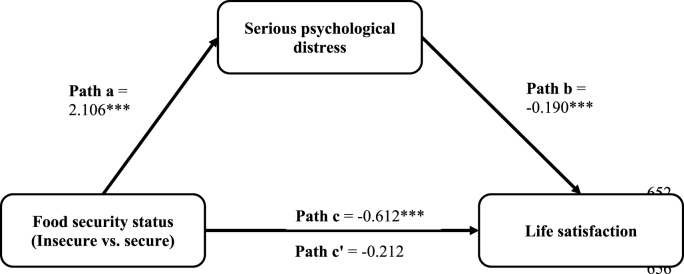
Regression coefficients for the mediation effects of serious psychological distress (SPD) on the relationship between food security status and life satisfaction, adjusting for employment, healthcare utilization, and sociodemographic characteristics among immigrant adults. The coefficients are statistically significant at *p < 0.05. **p < 0.01. ***p < 0.001. Path a represents the effect of food insecurity (vs. food security) on SPD. Path b represents the effect of SPD on life satisfaction, controlling for the effect of food insecurity. Path c represents the total (mediated and direct) effect of food insecurity (vs. food security) on life satisfaction, not accounting for the effect of SPD. Finally, Path c' represents the direct effects of food insecurity (vs. food security) on life satisfaction, after accounting for the effect of SPD. The product of Path a and Path b represents the indirect (mediated) effect (a*b = −0.400***) of food insecurity on life satisfaction through SPD.

As shown in Table 2, food insecurity (versus security) was positively associated with SPD (a = 2.106, *p* < 0.001), while SPD was negatively associated with life satisfaction (b = −0.190, *p* < 0.001), adjusting for the confounders or covariates (see Table S1 in the supplemental file for the results of the covariates). There was a negative indirect effect (a*b = −0.400, *p* < 0.001) and a total effect (c = −0.612, *p* < 0.001) of food insecurity on life satisfaction. The direct effect shows that SPD partially mediates the relationship between food insecurity and life satisfaction. The proportion of total effect that is mediated is 0.654 (i.e., −0.400/−0.612 = 0.6536), suggesting that the effect of food insecurity is reduced by about 65.40% after accounting for SPD. In other words, approximately 65.40% of the effect of food insecurity on life satisfaction is explained by the indirect effect of food security on SPD. Fig. 2 represents the path diagram illustrating the mediation effects (including the significance levels).

**Fig. 3:**
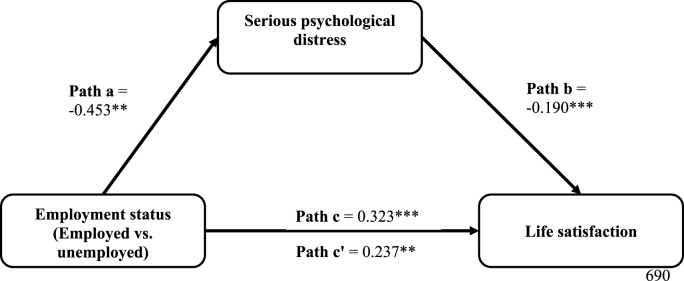
Regression coefficients for the mediation effects of serious psychological distress (SPD) on the relationship between employment status and life satisfaction, adjusting for food security status, healthcare utilization, and sociodemographic characteristics among immigrant adults. The coefficients are statistically significant at *p < 0.05. **p < 0.01. ***p < 0.001. Path a represents the effect of being employed (vs. unemployed) on SPD. Path b represents the effect of SPD on life satisfaction, controlling for the effect of being employed. Path c represents the total (mediated and direct) effect of being employed (vs. unemployed) on life satisfaction, not accounting for the effect of SPD. Finally, Path c’ represents the direct effects of being employed (vs. unemployed) on life satisfaction, after accounting for the effect of SPD. The product of Path a and Path b represents the indirect (mediated) effect (a*b = 0.086**) of employment status on life satisfaction through SPD.

Table 3 shows that being employed (versus unemployed) was negatively associated with SPD (a = −0.453, *p* < 0.01), and SPD was negatively associated with life satisfaction (b = −0.190, *p* < 0.001). Statistically significant positive indirect (a*b = 0.086, *p* < 0.01), direct (c' = 0.237, *p* < 0.01) and total effects (c = 0.323, *p* < 0.001) of being employed on life satisfaction were found. That is, the effect of employment is reduced by about 26.60% after accounting for SPD. The significant direct effect indicates that SPD partially mediates the association between employment and life satisfaction. Table S2 in the supplemental file presents the results for the covariates adjusted in the model. Fig. 3 further illustrates the mediation effects (including the significance levels).

**Fig. 4:**
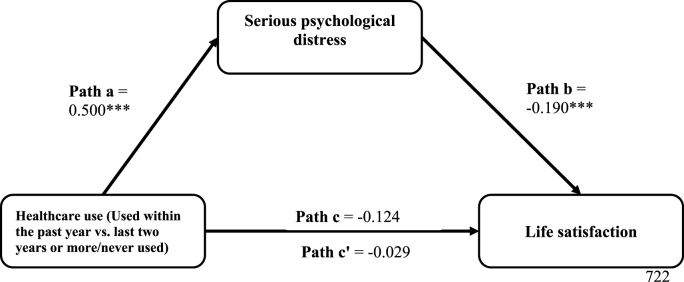
Regression coefficients for the mediation effects of serious psychological distress (SPD) on the relationship between healthcare utilization status and life satisfaction, adjusting for food security status, employment status, and sociodemographic characteristics among immigrant adults. The coefficients are statistically significant at *p < 0.05. **p < 0.01. ***p < 0.001. Path a represents the effect of healthcare utilization within the past year (vs. more than a year) on SPD. Path b represents the effect of SPD on life satisfaction, controlling for the effect of healthcare utilization within the past year. Path c represents the total (mediated and direct) effect of healthcare utilization within the past year (vs. more than a year) on life satisfaction, not accounting for the effect of SPD. Finally, Path c’ represents the direct effects of healthcare utilization within the past year (vs. more than a year) on life satisfaction, after accounting for the effect of SPD. The product of Path a and Path b represents the indirect (mediated) effect (a*b = −0.095**) of healthcare utilization status on life satisfaction through SPD.

In Table 4, healthcare utilization within the past 12 months (versus more than 12 months/never) was significantly positively associated with SPD (a = 0.500, *p* < 0.001), while SPD was negatively associated with life satisfaction (b = −0.190, *p* < 0.001). Healthcare utilization within the past 12 months had a significant negative indirect effect on life satisfaction (a*b = −0.095, *p* < 0.01). The direct and total effects were not statistically significant. The lack of significant direct effect suggests that SPD almost fully mediates the association between healthcare utilization in the past 12 months and life satisfaction. The results of the covariates in the model are presented in Table S3 in the supplemental file. The visual representation of the mediation effects (including the significance levels) is presented in Fig. 4.

## 4 Discussion

In a large, nationally representative sample of immigrant adults in the U.S., we evaluated the mediation effects of mental health (i.e., psychological distress) on the relationship between social needs and life satisfaction. We observed that psychological distress significantly mediated the effects of food security, employment, and healthcare utilization on life satisfaction among the immigrants, respectively. Social needs influenced psychological distress, which in turn, influenced life satisfaction. Thus, the effects of these social needs on life satisfaction have reduced upon accounting for psychological distress. Notably, we found that immigrants with food insecurity (versus food security) or who used healthcare within the past 12 months (versus more than 12 months/never) had increased risks of psychological distress and lower life satisfaction, while those who had employment (versus unemployment) had decreased psychological distress and increased life satisfaction. Increased psychological distress, on the other hand, decreased life satisfaction. The mediation effects suggest that individuals with food insecurity and those who used healthcare within the past 12 months further had decreased life satisfaction, whereas the positive effects of employment also decreased when considering the effect of psychological. These findings underscore the need to consider mental health in interventions aimed at improving social needs to enhance immigrants’ life satisfaction. The interventions may be more beneficial by improving the pathways linking social needs to life satisfaction with an integral effort to address mental health symptoms or disorders.

Our findings are consistent with past studies on the adverse effects of poor socioeconomic conditions or social needs on poor mental health and lower life satisfaction, as well as poor mental health reducing life satisfaction among immigrants (Berggren et al., 2020; Calvo et al., 2017, 2019; Chen & Hou, 2019; Derr, 2016; Guo & Cheng, 2017; Harrelson, 2019; Kim et al., 2014, Kim et al., 2021, Kim et al., 2021; Lombardo et al., 2018; Park et al., 2012; Pourmotabbed et al., 2020; Salahodjaev & Mirziyoyeva, 2021). Yet the past studies did not examine the impact of the intersection of social needs and mental health on life satisfaction among immigrants in the U.S. The results are of public health significance because public health professionals and researchers might consider life satisfaction intervention or policy effects, and the study estimates they might expect when considering how improving socioeconomic conditions or social needs and mental health could enhance life satisfaction. The life satisfaction interventions may include improving psychological resilience (e.g., social support, culturally affirming, and positive attitude) and coping mechanisms (e.g., stress management, exercise, and time management) that are useful in enhancing mental health and problem-solving strategies to thrive in the face of adversity, as well as offset the risk factors for mental health and socioeconomic challenges (Algorani & Gupta, 2022; Srivastava, 2011).

Mental health-related potential links between social needs and life satisfaction among immigrants may require the consideration of three dynamics (Baron & Kenny, 1986; Berggren et al., 2020; Calvo et al., 2019; Derr, 2016; Dou et al., 2022; Kubzansky et al., 2018; Lombardo et al., 2018; Pourmotabbed et al., 2020): (1) improvement of socioeconomic or social need resources that reduce mental health symptoms or disorders; (2) indirect effects of social needs through mental health, and (3) direct effects of social needs through mental health pathways. When considering resources to improve social needs, it is essential to note that immigrants with poor socioeconomic conditions or social needs have high psychological distress, which, in turn, decreases their life satisfaction. A possible explanation for these findings is the complexity of immigrants' circumstances in their host countries. Although the socioeconomic conditions of immigrants in the host countries are often better than their countries of origin, they are also burdened with limited socioeconomic opportunities (e.g., food security, employment, healthcare access, and housing) and poor mental health in their host countries (Brydsten et al., 2019; Calvo et al., 2019; Dhokarh et al., 2011; Dou et al., 2022; Garasky et al., 2006; Iglesias-Rios et al., 2015). Immigrants have elevated risks of food insecurity, unemployment or stable employment, poor mental health, and lack of healthcare utilization, including mental health services, than their nonimmigrant counterparts (Calvo et al., 2019; Derr, 2016; Dhokarh et al., 2011; Dou et al., 2022; Garasky et al., 2006; Guo & Cheng, 2017). They have higher risks of mental health problems but are less likely to use mental health services (Derr, 2016). Difficulties in acculturation, such as language barriers, may limit opportunities for immigrants to secure appropriate employment that matches their qualifications in the host-culture environment (Dhokarh et al., 2011; Dou et al., 2022; Iglesias-Rios et al., 2015). Additionally, both undocumented and documented individuals may fear immigration authorities due to the risk of deportation (e.g., Public Charge Rule, which prohibits lawful permanent residents from sponsoring family members) (Ormiston et al., 2023). Lack of access to healthcare services and governmental assistance programs (e.g., food and welfare assistance programs) due to ineligibility are also plausible reason for limited opportunities among immigrants (Dhokarh et al., 2011; Dou et al., 2022; Iglesias-Rios et al., 2015). Reducing household food insecurity and hunger, unemployment, and mental health disorders are some of the specific objectives of Healthy People 2030 in improving overall health and well-being, especially among minority populations (Ochiai et al., 2021; Office of Disease Prevention & Health Promotion, 2023; Pronk et al., 2021). Therefore, identifying specific mechanisms linking these social needs to life satisfaction can augment efforts in achieving the specific objectives of boosting immigrants’ life satisfaction and well-being.

Our study has several strengths, including using a large, nationally representative sample of immigrants, validated measures, and robust statistical methods among an understudied population. However, it also has some limitations that need to be noted. Despite consideration of the temporal sequence of the factors, our results were based on analysis of cross-sectional data, which could not be used to make causal inferences because reverse or bidirectional relationships could be possible. Hence, we did not intend to make any causal inferences in this study. Future longitudinal or experimental research could evaluate the causal relationships. Another limitation is that the independent and dependent variables were based on self-response, a potential bias that could lead to underestimation of effects or health behavior. While we used validated subjective measures, future studies may use objective health measures to reduce this potential self-response bias. Based on previous studies, we adjusted for potential confounders, but residual confounders could have also influenced the results. The immigrants' cultural context (e.g., language, country of origin) and circumstances of migration (e.g., war, religious persecution) might be some residual confounders that could affect the assessment of mental health and life satisfaction and, as such, could be included in future studies.

## 5 Conclusions

Our findings indicate that psychological distress mediates the effect of food insecurity, past 12 months of healthcare utilization, and employment on life satisfaction among immigrant adults. More specifically, psychological distress partially mediated the relationship between food insecurity or employment and life satisfaction, while the relationship between the past 12 months of healthcare utilization and life satisfaction was almost fully mediated by psychological distress. The findings highlight the need to improve these social needs and mental health to enhance life satisfaction among immigrants as part of the specific objectives of Healthy People 2030 in advancing overall health and well-being among minority populations (Ochiai et al., 2021; Office of Disease Prevention & Health Promotion, 2023; Pronk et al., 2021). More efforts toward developing culturally and linguistically appropriate social and mental health interventions may augment existing interventions and policies aimed at increasing access to food security, employment opportunities, and healthcare and mental health services in conducive living environments for immigrant populations. Specific interventions should include increasing awareness and access to services like translation/interpretation, medical/mental health, education/training, job fair, social assistance programs, food, childcare, transportation, and housing services. These services may also be provided in immigrant communities to enhance access and utilization among the immigrants. Culturally appropriate and adequate nutritious food, for instance, can be more socially acceptable in immigrant communities than food that do not meet their cultural needs (Pourmotabbed et al., 2020). Using culturally competent service providers who are trained in providing services in immigrant communities can facilitate rapport building with immigrants, service provision and utilization, and long-term trust in services. In summary, this study has determined the pathways linking social needs to life satisfaction that public health professionals and policymakers might consider in developing policies and interventions to improve life satisfaction. These cross-sectional findings further serve as a foundation for researchers to consider in developing longitudinal studies to evaluate the observed pathways.

## Author credit statement

**David Adzrago:** Conceptualization, Methodology, Data Curation, Formal Analysis, Validation, Visualization, Writing – Original Draft Preparation, Writing − Review & Editing. **Faustine Williams**: Conceptualization, Methodology, Visualization, Resources, Writing − Review & Editing, Supervision.

## Financial disclosures

None.

## Declaration of competing interest

None.

## Data Availability

Data are publicly available at https://www.cdc.gov/nchs/nhis/2021nhis.htm
