# Supplementary material for: Mediation analysis of mental health characteristics linking social needs to life satisfaction among immigrants
Source: SSM Popul Health. 2023 Oct 4;24:101522. doi: 10.1016/j.ssmph.2023.101522 (PMC10563063; doi:10.1016/j.ssmph.2023.101522)
Supplement: Multimedia component 1 [file mmc1.docx]

**Table S1.** The results of factors adjusted in the model (Table 2) evaluating the mediation effects of SPD on the relationship between food security and life satisfaction.

|  |  | **SPD (M)** | |  | **Life satisfaction (Y)** | |
| --- | --- | --- | --- | --- | --- | --- |
|  |  | **Coeff. (SE)** | **95% CI** |  | **Coeff. (SE)** | **95% CI** |
| **Age groups** |  |  |  |  |  |  |
| 18-25 |  | Ref |  |  | Ref |  |
| 26-34 |  | -0.297 (0.260) | (-0.809, 0.214) |  | 0.051 (0.168) | (-0.279, 0.381) |
| 35-49 |  | -0.572* (0.237) | (-1.038, -0.106) |  | -0.033 (0.161) | (-0.349, 0.282) |
| 50-64 |  | -0.230 (0.254) | (-0.728, 0.269) |  | -0.167 (0.162) | (-0.485, 0.152) |
| 65 and older |  | -0.589* (0.292) | (-1.164, -0.015) |  | -0.113 (0.175) | (-0.458, 0.231) |
| **Sex** |  |  |  |  |  |  |
| Male |  | Ref |  |  | Ref |  |
| Female |  | 0.444** (0.131) | (0.186, 0.702) |  | 0.156* (0.069) | (0.021, 0.291) |
| **Sexual orientation** |  |  |  |  |  |  |
| Heterosexual |  | Ref |  |  | Ref |  |
| Gay/Lesbian |  | 2.173** (0.733) | (0.733, 3.613) |  | -0.018 (0.214) | (-0.438, 0.402) |
| Bisexual |  | 3.155** (1.002) | (1.187, 5.124) |  | -0.330 (0.316) | (-0.951, 0.292) |
| Other/uncertain |  | 0.961 (0.513) | (-0.047, 1.968) |  | -0.132 (0.231) | (-0.586, 0.321) |
| **Citizenship status** |  |  |  |  |  |  |
| Citizen |  | Ref |  |  | Ref |  |
| Non-citizen |  | 0.099 (0.148) | (-0.192, 0.389) |  | -0.015 (0.063) | (-0.138, 0.108) |
| **Race/ethnicity** |  |  |  |  |  |  |
| Non-H White |  | Ref |  |  | Ref |  |
| Non-H Black/African American |  | -0.813*** (0.223) | (-1.253, -0.374) |  | 0.140 (0.145) | (-0.146, 0.425) |
| Hispanic |  | -0.202 (0.163) | (-0.522, 0.119) |  | 0.390*** (0.081) | (0.230, 0.550) |
| Other/multi-racial |  | -0.369* (0.150) | (-0.663, -0.074) |  | -0.013 (0.077) | (-0.165, 0.139) |
| **Family income to poverty ratio** |  | -0.021 (0.020) | (-0.060, 0.019) |  | 0.014 (0.010) | (-0.005, 0.033) |
| **Social/emotional support frequency** |  |  |  |  |  |  |
| Always/usually/sometimes |  | Ref |  |  | Ref |  |
| Rarely/never |  | 0.308 (0.208) | (-0.101, 0.717) |  | -0.220 (0.115) | (-0.446, 0.007) |
| **Employment status** |  |  |  |  |  |  |
| Unemployed |  | Ref |  |  |  |  |
| Employed |  | -0.453** (0.167) | (-0.781, -0.124) |  | 0.237** (0.082) | (0.076, 0.398) |
| **Healthcare utilization** |  |  |  |  |  |  |
| Within the last two years or More/never used |  | Ref |  |  | Ref |  |
| Within the past year |  | 0.500*** (0.138) | (0.229, 0.770) |  | -0.029 (0.070) | (-0.167, 0.108) |

Regression coefficients are unstandardized. Standard errors (SEs) are in parentheses. Bootstrap sample size = 1000. 95% CI = confidence interval. Coeff. = regression coefficient. Ref = reference group. *p<0.05. **p<0.01. ***p<0.001. SPD = serious psychological distress. Non-H = non-Hispanic.

**Table S2.** The results of factors adjusted in the Model (Table 3) evaluating the mediation effects of SPD on the relationship between employment status and life satisfaction.

|  |  | **SPD (M)** | |  | **Life satisfaction (Y)** | |
| --- | --- | --- | --- | --- | --- | --- |
|  |  | **Coeff. (SE)** | **95% CI** |  | **Coeff. (SE)** | **95% CI** |
| **Age groups** |  |  |  |  |  |  |
| 18-25 |  | Ref |  |  | Ref |  |
| 26-34 |  | -0.297 (0.260) | (-0.809, 0.214) |  | 0.051 (0.168) | (-0.279, 0.381) |
| 35-49 |  | -0.572* (0.237) | (-1.038, -0.106) |  | -0.033 (0.161) | (-0.349, 0.282) |
| 50-64 |  | -0.230 (0.254) | (-0.728, 0.269) |  | -0.167 (0.162) | (-0.485, 0.152) |
| 65 and older |  | -0.589* (0.292) | (-1.164, -0.015) |  | -0.113 (0.175) | (-0.458, 0.231) |
| **Sex** |  |  |  |  |  |  |
| Male |  | Ref |  |  | Ref |  |
| Female |  | 0.444** (0.131) | (0.186, 0.702) |  | 0.156* (0.069) | (0.021, 0.291) |
| **Sexual orientation** |  |  |  |  |  |  |
| Heterosexual |  | Ref |  |  | Ref |  |
| Gay/Lesbian |  | 2.173** (0.733) | (0.733, 3.613) |  | -0.018 (0.214) | (-0.438, 0.402) |
| Bisexual |  | 3.155** (1.002) | (1.187, 5.124) |  | -0.330 (0.316) | (-0.951, 0.292) |
| Other/uncertain |  | 0.961 (0.513) | (-0.047, 1.968) |  | -0.132 (0.231) | (-0.586, 0.321) |
| **Citizenship status** |  |  |  |  |  |  |
| Citizen |  | Ref |  |  | Ref |  |
| Non-citizen |  | 0.099 (0.148) | (-0.192, 0.389) |  | -0.015 (0.063) | (-0.138, 0.108) |
| **Race/ethnicity** |  |  |  |  |  |  |
| Non-H White |  | Ref |  |  | Ref |  |
| Non-H Black/African American |  | -0.813*** (0.223) | (-1.253, -0.374) |  | 0.140 (0.145) | (-0.146, 0.425) |
| Hispanic |  | -0.202 (0.163) | (-0.522, 0.119) |  | 0.390*** (0.081) | (0.230, 0.550) |
| Other/Multi-Racial |  | -0.369* (0.150) | (-0.663, -0.074) |  | -0.013 (0.077) | (-0.165, 0.139) |
| **Family income to poverty ratio** |  | -0.021 (0.020) | (-0.060, 0.019) |  | 0.014 (0.010) | (-0.005, 0.033) |
| **Social/emotional support Frequency** |  |  |  |  |  |  |
| Always/usually/sometimes |  | Ref |  |  | Ref |  |
| Rarely/never |  | 0.308 (0.208) | (-0.101, 0.717) |  | -0.220 (0.115) | (-0.446, 0.007) |
| **Food security status** |  |  |  |  |  |  |
| Secure |  | Ref |  |  |  |  |
| Insecure |  | 2.106*** (0.365) | (1.389, 2.823) |  | -0.212 (0.157) | (-0.521, 0.097) |
| **Healthcare utilization** |  |  |  |  |  |  |
| Within the last two years or More/never used |  | Ref |  |  | Ref |  |
| Within the past year |  | 0.500*** (0.138) | (0.229, 0.770) |  | -0.029 (0.070) | (-0.167, 0.108) |

Regression coefficients are unstandardized. Standard errors (SEs) are in parentheses. Bootstrap sample size = 1000. 95% CI = confidence interval. Coeff. = regression coefficient. Ref = reference group. *p<0.05. **p<0.01. ***p<0.001. SPD = serious psychological distress. Non-H = non-Hispanic.

**Table S3.** The results of factors adjusted in the Model (Table 4) evaluating the mediation effects of SPD on the relationship between healthcare utilization and life satisfaction.

|  |  | **SPD (M)** | |  | **Life satisfaction (Y)** | |
| --- | --- | --- | --- | --- | --- | --- |
|  |  | **Coeff. (SE)** | **95% CI** |  | **Coeff. (SE)** | **95% CI** |
| **Age groups** |  |  |  |  |  |  |
| 18-25 |  | Ref |  |  | Ref |  |
| 26-34 |  | -0.297 (0.260) | (-0.809, 0.214) |  | 0.051 (0.168) | (-0.279, 0.381) |
| 35-49 |  | -0.572* (0.237) | (-1.038, -0.106) |  | -0.033 (0.161) | (-0.349, 0.282) |
| 50-64 |  | -0.230 (0.254) | (-0.728, 0.269) |  | -0.167 (0.162) | (-0.485, 0.152) |
| 65 and older |  | -0.589* (0.292) | (-1.164, -0.015) |  | -0.113 (0.175) | (-0.458, 0.231) |
| **Sex** |  |  |  |  |  |  |
| Male |  | Ref |  |  | Ref |  |
| Female |  | 0.444** (0 .131) | (0.186, 0.702) |  | 0.156* (0.069) | (0.021, 0.291) |
| **Sexual orientation** |  |  |  |  |  |  |
| Heterosexual |  | Ref |  |  | Ref |  |
| Gay/Lesbian |  | 2.173** (0.733) | (0.733, 3.613) |  | -0.018 (0.214) | (-0.438, 0.402) |
| Bisexual |  | 3.155** (1.002) | (1.187, 5.124) |  | -0.330 (0.316) | (-0.951, 0.292) |
| Other/uncertain |  | 0.961 (0.513) | (-0.047, 1.968) |  | -0.132 (0.231) | (-0.586, 0.321) |
| **Citizenship status** |  |  |  |  |  |  |
| Citizen |  | Ref |  |  | Ref |  |
| Non-citizen |  | 0.099 (0.148) | (-0.192, 0.389) |  | -0.015 (0.063) | (-0.138, 0.108) |
| **Race/ethnicity** |  |  |  |  |  |  |
| Non-H White |  | Ref |  |  | Ref |  |
| Non-H Black/African American |  | -0.813*** (0.223) | (-1.253, -0.374) |  | 0.140 (0.145) | (-0.146, 0.425) |
| Hispanic |  | -0.202 (0.163) | (-0.522, 0.119) |  | 0.390*** (0.081) | (0.230, 0.550) |
| Other/multi-racial |  | -0.369* (0.150) | (-0.663, -0.074) |  | -0.013 (0.077) | (-0.165, 0.139) |
| **Family income to poverty ratio** |  | -0.021 (0.020) | (-0.060, 0.019) |  | 0.014 (0.010) | (-0.005, 0.033) |
| **Social/emotional support frequency** |  |  |  |  |  |  |
| Always/usually/sometimes |  | Ref |  |  | Ref |  |
| Rarely/never |  | 0.308 (0.208) | (-0.101, 0.717) |  | -0.220 (0.115) | (-0.446, 0.007) |
| **Food security status** |  |  |  |  |  |  |
| Secure |  | Ref |  |  |  |  |
| Insecure |  | 2.106*** (0.365) | (1.389, 2.823) |  | -0.212 (0.157) | (-0.521, 0.097) |
| **Employment status** |  |  |  |  |  |  |
| Unemployed |  | Ref |  |  | Ref |  |
| Employed |  | -0.453** (0.167) | (-0.781, -0.124) |  | 0.237** (0.082) | (0.076, 0.398) |

Regression coefficients are unstandardized. Standard errors (SEs) are in parentheses. Bootstrap sample size = 1000. 95% CI = confidence interval. Coeff. = regression coefficient. Ref = reference group. *p<0.05. **p<0.01. ***p<0.001. SPD = serious psychological distress. Non-H = non-Hispanic.
